# *De novo* transcriptome assembly for four species of crustose coralline algae and analysis of unique orthologous genes

**DOI:** 10.1038/s41598-019-48283-1

**Published:** 2019-08-30

**Authors:** Tessa M. Page, Carmel McDougall, Guillermo Diaz-Pulido

**Affiliations:** 1 School of Environment and Science, Nathan Campus, Griffith University, 170 Kessels Road, Nathan, QLD 4111 Australia; 2 Australian Rivers Institute, Nathan Campus, Griffith University, 170 Kessels Road, Nathan, QLD 4111 Australia

**Keywords:** Transcriptomics, Ecology

## Abstract

Crustose coralline algae (CCA) are calcifying red macroalgae that reef build in their own right and perform essential ecosystem functions on coral reefs worldwide. Despite their importance, limited genetic information exists for this algal group. *De novo* transcriptomes were compiled for four species of common tropical CCA using RNA-seq. Sequencing generated between 66 and 87 million raw reads. Transcriptomes were assembled, redundant contigs removed, and remaining contigs were annotated using Trinotate. Protein orthology analysis was conducted between CCA species and two noncalcifying red algae species from NCBI that have published genomes and transcriptomes, and 978 orthologous protein groups were found to be uniquely shared amongst CCA. Functional enrichment analysis of these ‘CCA-specific’ proteins showed a higher than expected number of sequences from categories relating to regulation of biological and cellular processes, such as actin related proteins, heat shock proteins, and adhesion proteins. Some proteins found within these enriched categories, i.e. actin and GH18, have been implicated in calcification in other taxa, and are thus candidates for involvement in CCA calcification. This study provides the first comprehensive investigation of gene content in these species, offering insights not only into the evolution of coralline algae but also of the Rhodophyta more broadly.

## Introduction

Crustose coralline algae (CCA) are calcifying red algae that form crusts on marine substrates worldwide, from polar regions to the tropics, and from intertidal zones to deep below the photic zone^1,2^. CCA are particularly abundant in tropical reefs, occupying much of the hard substrates within coral reef ecosystems^3^. Tropical CCA are key players in contributing to the global carbon cycle^4^ and provide various ecosystem functions, such as contributing to the structural complexity of coral reefs by building and cementing the carbonate framework^5^, and inducing the metamorphosis and settlement of coral larvae^6,7^ and other economically and ecologically important invertebrates^8^. Furthermore, CCA assist coral reefs to withstand and recover from disturbances^9^, and can therefore mitigate some of the negative impacts from the loss of reef structural complexity brought on by anthropogenic and naturogenic disruptions.

**Figure 1:**
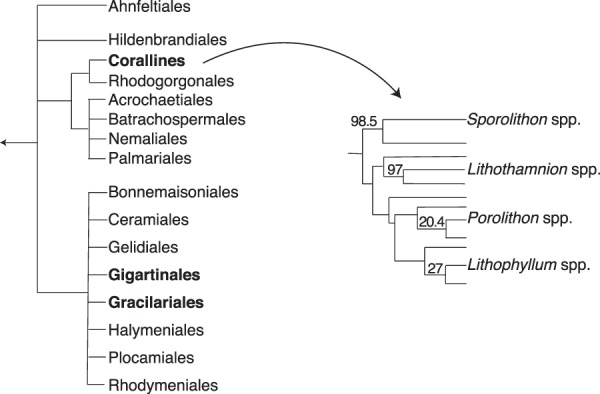
Phylogenetic tree for the phylum Rhodophyta. Inset depicts the relationship of CCA species from different orders within the Corallines group. Phylogenetic tree for Rhodophyta based on Freshwater *et al*.^79^, Pueschel 1994^80^, Ragan *et al*.^81^, Saunders and Bailey 1997^82^. Tree for CCA is adapted from Aguirre *et al*.^10^ & Rösler *et al*.^21^. Node labels on inset tree of CCA species depict evolutionary time in millions of years.

Coralline algae evolved from a red algal ancestor in the Early Cretaceous and began to diversify during the Early Miocene^10^ (Fig. 1). Coralline algae are unique amongst other red algae species due to the type of calcium carbonate used in this group^11^, and their ability to calcify within cell walls^12^. Coralline algae produce high magnesium calcite crystals generally oriented radially perpendicular to the cell wall as well as crystals oriented parallel to the wall in the interfilament region^12,13^, and their calcification process is considered to be an “organic matrix-mediated process^12^”. In contrast, the calcification process of other calcifying algae species, such as *Halimeda* spp., is considered to be “biologically induced”, occurring primarily outside the cell^12^. Calcification in coralline algae can be considered, to some extent, to be cellularly regulated, and is somewhat similar to the calcification process of coccolithophores which is thought to be an extreme example of “organic matrix-mediated” calcification or “biologically controlled” calcification^12,14^. Studies have also found the calcification process in coralline algae and coccolithophores to be linked to unusual polysaccharides^15^. The production and maintenance of these calcified skeletons is what allows CCA to play such a crucial role across tropical coral reefs and sets them apart from other calcifying algal species^16^. Coralline algae have been found to also upregulate the pH of their calcifying fluid/medium during calcification^17^. Due to the uniqueness of calcification in coralline algae, and their importance in general, there is a need for molecular mechanistic studies on coralline algae^4^. The use of molecular studies could lead to an understanding of the independent evolution of calcification in coralline algae, as well as an understanding of how these calcified organisms persisted and diversified during times of previously high *p*CO_2_ and temperature^18^.

An appreciation of the importance of CCA and the services they provide, and their sensitivity to climate change impacts, has led to a number of studies into their biology^19^, calcification^17,20^, phylogeny^18,21^, and physiology^22,23^. However limited molecular information (genomes, transcriptomes, gene expression profiles, or proteomes) exists for these species. This knowledge gap greatly limits our ability to fully understand whole organism function and the responses of this group of red algae to pressing environmental issues, such as changes in their environment brought on by human-induced change (e.g. ocean acidification and warming or declining water quality). The sequencing of genomes and/or transcriptomes provides essential information for the elucidation of the mechanisms that underpin physiological and biological traits and responses. Although sequencing has become more readily accessible^24,25^, genomes and annotated transcriptomes for many environmentally and economically important species are unavailable. ‘Omics’ studies are lacking in algae in general^26^, with minimal genomic information available in algae when compared to land plants^27^ or other phyla. Only 51 whole genomes are available for green algae, 3 for brown algae, and 9 for red algae, compared to the 672 for chordates, the 400 for arthropods, and the 345 for vascular plants (Table 1). A similar trend is seen for transcriptomes (Table 1). For coralline algae no complete genomes or transcriptomes have been published, however, mitochondrial^28–30^ and plastid genomes^31^ have been sequenced for some species. Therefore, there is a major knowledge gap in our understanding of the molecular landscape of coralline algae.

**Table 1:** Number of published whole genomes and assembled transcriptomes for different taxa.

	Whole Genomes	Assembled Transcriptomes
Chordates	672	642
Arthropods	400	1442
Vascular plants	345	877
Green Algae	51	20
Brown Algae	3	42
Red Algae	9	36
Coralline Algae	0	0

Data taken from NCBI, as of February 2019. Transcriptome data taken from NCBI’s Transcriptome Shotgun Assembly Sequence Database and is most likely an overestimate as duplication of species is not taken into account.

In the present study, we generated transcriptomes for four species of tropical CCA: *Porolithon* cf. *onkodes*, *Sporolithon* cf. *durum*, *Lithothamnion* cf. *proliferum*, and *Lithophyllum* cf. *insipidum*, hereinafter referred to as *Porolithon*, *Sporolithon*, *Lithothamnion*, and *Lithophyllum*, respectively. These species were collected in the Great Barrier Reef, Australia, and were selected because they are common and abundant in tropical reefs and belong to different evolutionary lineages in the coralline algae (*sensu lato*). To ensure inclusion of stress-response genes within our transcriptomes we included samples taken after exposing the CCA to combined and independent increased temperature and decreased pH treatments. Orthology inferences were conducted to identify putative orthologous genes between these CCA species and other red algae for which genomic or transcriptomic data was available. A number of orthogroups appear unique to CCA, and inference of the likely functions of these genes provides insight into the evolution of these important reef-builders. This study provides a valuable framework for understanding the molecular biology of CCA and insight into genes potentially involved in important processes in CCA, such as calcification. Additionally, we hope this study will facilitate future research into the molecular responses and vulnerability of CCA to future environmental change.

## Results and Discussion

### *De novo* transcriptome assembly

The current study presents novel transcriptome assemblies for four species of crustose coralline algae (CCA): *Sporolithon*, *Lithothamnion*, *Porolithon*, and *Lithophyllum*. RNA-seq libraries for each species were generated from pooled RNA extracted from one individual reproductive adult from each of the different treatment conditions (n = 6) (refer to Methods & Supplementary Methods 1). Sequencing of these libraries produced between 66–87 million paired-end reads per species (*n* = 4). Variability was found across species when comparing assembly statistics (Table 2). *Lithothamnion* contained the fewest raw reads, which equated into the fewest assembled transcripts. *Lithophyllum* had a similar number of raw reads to *Lithothamnion* but had significantly more assembled transcripts. *Sporolithon* and *Porolithon* were most similar in their number of raw reads and assembled transcripts in comparison to the other two species. Clustering of redundant transcripts using CD-Hit reduced contig numbers by 16–27% (Table 2). Mean contig length was not significantly different between species, but was variable, ranging from 498–694 base pairs (bp), and N50 values ranged between 602–1147 bp (Table 2). These assembly statistics are comparable to those of transcriptomes from other red algal species such as *Pyropia seriata*^32^, *Porphyra umbilicalis*^33^, and *Porphyra purpurea*^33^, except the assembly of *Lithophyllum* which had a much lower N50 value indicating a more fragmented assembly. Read representation within each assembly, assessed by mapping raw reads for each species against their respective *de novo* transcriptomes (RMBT%), was high (94% or above). Variability in number of assembled transcripts and their resulting summary statistics may be due to collection of individual crusts at different reproductive stages for each species. *Lithophyllum* is reproductive year-round, and the samples taken likely possessed numerous gametangial and/or tetrasporangial conceptacles. *Sporolithon* and *Porolithon* were just coming into their reproductive time of year and likely had fewer reproductive structures, whereas *Lithothamnion* probably had the fewest number of reproductive structures as it has been found to be primarily reproductive in summer months (pers. obs.).

**Table 2:** Summary statistics for *Sporolithon*, *Porolithon*, *Lithothamnion*, and *Lithophyllum de novo* transcriptome assemblies.

	Sporolithon	Porolithon	Lithothamnion	Lithophyllum
Raw reads	81,176,680	87,281,439	66,119,011	64,651,990
Contigs, with Jaccard Clip	231,324	163,784	54,557	306,668
Contigs after CDHIT clustering	185,481	118,126	45,633	233,751
Mean length (bp)	589.1	584.72	693.59	498
N50 (bp)	862	788	1,147	602
RMBT%	95.22%	97.25%	98.68%	94.07%
GC%	42.42%	44.53%	49.02%	46.92%

N50 statistic denotes the length of contigs which cover 50% of the transcriptome. RMBT% is the percentage of reads that mapped back to the transcriptome. GC% represents the percentage or content of guanine-cytosine within the transcriptome.

### Quality assessment of transcriptomes

The quality of each transcriptome was assessed using the Benchmarking Universal Single-Copy Ortholog (BUSCO) assessment tool^34^. CCA *de novo* transcriptomes were compared against whole genome protein data from the noncalcified red algal species, *Chondrus crispus* and *Gracilariopsis chorda*, from the orders Gigartinales and Gracilariales, respectively (Fig. 1). BUSCO analysis of the four CCA transcriptomes showed that, out of the 303 near-universal single-copy eukaryote orthologs, between 87% and 93% complete sequences and 4% to 7% fragmented or partial sequences were detected (Table 3). Additionally, only between 3% and 7% of near-universal genes were classified as missing in the CCA transcriptomes, indicating high quality and good coverage. BUSCO analysis run on the reference genome proteins of the two noncalcifying red algae species returned similar measures of completeness, however, as expected for a curated genomic dataset, had a higher percentage of complete single-copy orthologs (73.90% to 88.80%), and a lower percent of duplication (2.60% to 3%). A higher percentage of missing BUSCOs was found in *C*. *crispus* (15.60%) when compared to the four CCA species, whereas, *G*. *chorda* had a similar percentage missing as *Lithophyllum* (Table 3). Three BUSCOs, EOG09370A22 (encoding a glycosyl transferase), EOG09370KWF (encoding a Per1-like gene), and EOG09370VTP (encoding a GPI mannosyltransferase) were found to be missing across all red algal species compared here, including the four CCA species. The absence of these genes from the transcriptomes of the 4 CCA species and the genomes of the 2 noncalcified red algal species may indicate that these genes were lost in the evolution of Rhodophyta. Additionally, there was one BUSCO, EOG09370JW6, missing across all CCA species, but present in the other noncalcifying red algae species. This gene, encoding for an elongator complex protein 4 (ELP4), is highly conserved in eukaryotes. It is part of a multi-subunit complex that interacts with elongating RNA polymerase II and is believed to facilitate transcription^35^, additionally, ELP4 plays a role in transfer RNA (tRNA) modification^36^. The elongator complex has also been tied to development and responses to biotic and abiotic stresses in plants^35^. In another study examining the elongator function in *Arabidopsis thaliana*, it was suggested that the elongator complex could influence mechanisms that produce carbon assimilates and the importation of sucrose^37^. The absence of the gene in all CCA transcriptomes generated here may indicate that it has been lost from the Corallines lineage entirely. Although many physiological states were sampled when collecting data for CCA it is possible that some genes may be missing from these assembled transcriptomes. Determination of the complete gene complement of these species, and confirmation of proposed gene losses, will require whole genome sequencing approaches.

**Table 3:** BUSCO results from the *de novo* transcriptomes of four species of CCA compared to the whole genome data of two species of noncalcifying red algal species, *C*. *crispus* and *G*. *chorda*.

BUSCO statistic	Coralline				Non-coralline	
	*Sporolithon*	*Porolithon*	*Lithothamnion*	*Lithophyllum*	*C*. *crispus*	*G*. *chorda*
Complete BUSCOs	278 (92%)	265 (88%)	264 (87%)	281 (93%)	234 (77%)	278 (91 %)
Complete - single-copy BUSCOs	99 (33%)	185 (61%)	222 (73%)	110 (36%)	226 (75%)	269 (88%)
Complete - duplicated BUSCOs	179 (59%)	80 (26%)	42 (14%)	171 (56%)	8 (3%)	9 (3%)
Fragmented BUSCOs	15 (5%)	17 (6%)	22 (7%)	11 (4%)	25 (8%)	10 (3%)
Missing BUSCOs	10 (3%)	21 (7%)	17 (6%)	11 (4%)	44 (15%)	15 (4%)

### Orthofinder analysis

Orthofinder was used to perform protein orthology analysis across red algae species using predicted proteins from the CCA transcriptomes generated here as well as those from the publicly available genomes of *C*. *crispus* and *G*. *chorda*. Given the high number of duplicated transcripts identified within transcriptomes via the BUSCO analysis, identification of orthologous sequences was conducted using translations of the single longest isoform of each Trinity gene from respective CCA transcriptomes. This resulted in a dataset that was less redundant, but also less complete (5% to 11% missing BUSCOs from this dataset compared with 3% to 7% previously).

**Figure 2:**
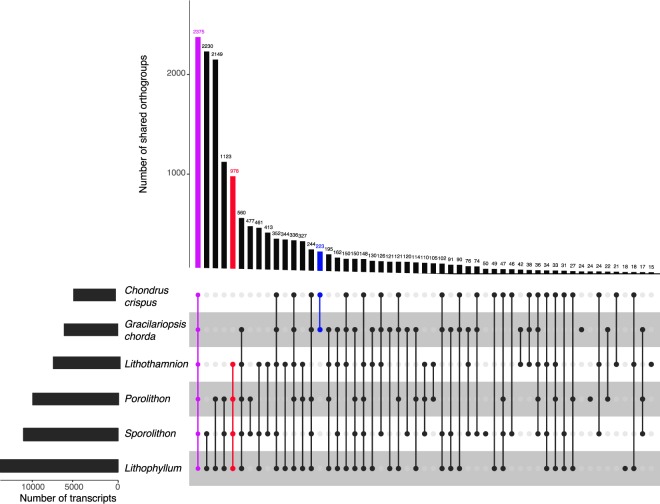
Plot visualising shared and unique orthologous protein groups across four species of crustose coralline algae, *Lithophyllum*, *Sporolithon*, *Porolithon*, and *Lithothamnion*, and two species of noncalcified red algae, *G*. *chorda* and *C*. *crispus*. Plot at the top shows the number of orthogroups found in the species indicated in the schematic below. Shared orthogroups across all red algae species are highlighted in purple, across only CCA species in red, and across only the noncalcifying red algal species in blue. Orthologous proteins were identified with Orthofinder and plotted using the R package, UpsetR^75^. Any relationships with <15 orthogroups were omitted.

2,375 orthogroups (genes derived from a common ancestral gene) were shared across all red algal species examined in this study (Fig. 2). 978 orthogroups were found to be CCA-specific, whereas only 223 were found to be shared between the other red algal species, *C*. *crispus* and *G*. *chorda*, to the exclusion of CCA. The low number of unique orthogroups found between these two species of fleshy macroalgae may relate to their phylogenetic relationship (these two species are from different, distantly related orders, whereas the CCA species are from closely related orders), or may reflect the use of protein sequences derived from transcriptomes vs proteins predicted from whole genome data. *Lithophyllum* had the most orthogroups in common with other species of CCA, this is probably due to the larger number of transcripts in the *Lithophyllum* dataset, whereas *Lithothamnion* had the fewest transcripts and therefore had fewer orthogroups common with the other species (Fig. 2).

### Enrichment analysis

#### Enriched CCA-specific genes

The genes found within the CCA-specific orthogroups are likely novel to the CCA lineage or represent expansions and diversification of ancestral algal gene families, and potentially reflect unique aspects of CCA biology with respect to noncalcified red algae. To evaluate these further an analysis was conducted to detect enrichment of putative functional categories within these CCA-specific orthogroups. Gene Ontology (GO) category enrichment was assessed against the whole transcriptome annotation, obtained from Trinotate v 3.1.1^38^. Overrepresentation of ‘biological process’ GO categories within the CCA-specific orthogroups was assessed via the Cytoscape^39^ plugin BiNGO^40^ using the complete transcriptome annotation for each CCA species as reference. Orthogroups that were found to be significantly (p < 0.01) overrepresented were further examined by extracting sequences from these families and submitting them to BLASTP (NCBI), using an e-value cutoff of 1e^−3^, to identify conserved domains and to indicate possible protein function (hypothesised via homology). GO categories such as the ‘regulation of transport’, ‘adhesion’, ‘supramolecular fibre organisation’, ‘regulation of actin cytoskeleton organisation’, and ‘regulation of cellular component organisation’ were found to be enriched in CCA-specific orthogroups (Table 4). A number of these categories were related (e.g., several involve actin) and are the result of enriched genes having multiple functional annotations. Only genes that had significant BLAST hits to other proteins were investigated further, as many of the genes within these categories appeared to be unique to CCA. It is notable that no categories related to biomineralisation or calcification were found to be enriched; this may reflect a different mode of biomineralisation of CCA to that of other, better studied organisms (e.g., polysaccharide mediated mineralisation as opposed to the protein-based matrix mediated mineralisation of vertebrates, molluscs and echinoderms). Although many of the genes that fell within these functionally enriched categories produced no significant hits in BLAST searches against NCBI’s nr database (and likely represent CCA-specific genes, or, possibly, contamination), a number appear to be members of larger gene families. Phylogenetic analysis was performed on these genes to provide further insight into their evolution and potential function.

**Table 4:** GO categories enriched across all CCA-specific orthogroups. Values represent adjusted *p* value (<0.01).

GO Term	Category Description	Sporolithon	Porolithon	Lithothamnion	Lithophyllum
GO:0032271	Regulation of protein polymerisation	2.08E-04	1.16E-04	5.07E-07	1.59E-04
GO:0030833	Regulation of actin filament polymerisation	1.13E-03	7.65E-03	6.85E-07	1.06E-04
GO:0030036	Actin cytoskeleton organisation	4.76E-04	1.33E-06	1.42E-06	7.13E-06
GO:0097435	Supramolecular fibre organisation	4.76E-04	1.14E-04	1.66E-06	8.87E-06
GO:0032956	Regulation of actin cytoskeleton organisation	6.05E-03	1.33E-06	1.06E-04	1.92E-04
GO:0043254	Regulation of protein complex assembly	4.45E-03	1.04E-04	3.29E-06	9.11E-03
GO:0051493	Regulation of cytoskeleton organisation	4.76E-04	2.71E-05	6.82E-06	3.39E-04
GO:0051128	Regulation of cellular component organisation	9.94E-05	2.96E-04	6.82E-06	3.25E-03
GO:0007010	Cytoskeleton organisation	1.52E-04	1.70E-14	9.90E-06	6.59E-24
GO:0051049	Regulation of transport	2.08E-04	2.37E-03	5.18E-06	2.18E-09
GO:0007155	Adhesion	9.09E-03	1.11E-05	2.94E-05	1.51E-15
GO:0030029	Actin filament-based process	1.13E-03	1.81E-04	1.99E-06	1.02E-05

#### ‘Regulation of transport’ related genes

**Figure 3:**
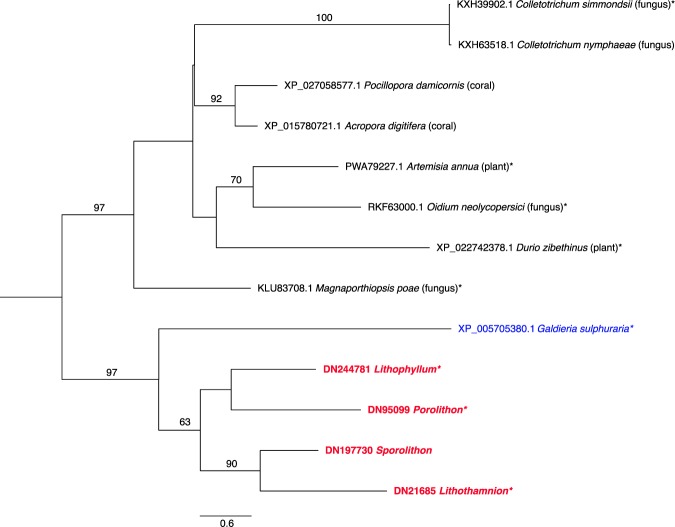
Best scoring maximum likelihood phylogenetic analysis of zinc finger domain sequences. Midpoint rooted tree, with bootstrap values <50 removed. Scale bar indicates the branch length for 0.6 amino acid (aa) substitutions. *Denotes full sequences that contain the CCHC-type zinc-finger like conserved domain. CCA names are shown in bolded red and other noncalcifying red algae species are in blue.

There were multiple orthogroups within the ‘regulation of transport’ category that were found to be overrepresented across the coralline species. One of these orthogroups contained sequences that contained a zinc finger domain, a domain previously undescribed in corallines. Zinc finger-like proteins have been found in other red algae species, such as the extremophilic unicellular species *Galdieria sulphuraria* (BioProject: PRJNA13023)^41^, *Cyanidioschyzon merolae* (BioProject: PRJNA28057)^42^, and *C*. *crispus*^43^. A phylogenetic tree was constructed for zinc finger type proteins, revealing that the CCA protein is most similar to a CCHC-type zinc finger domain protein found in *G*. *sulphuraria* and that proteins of this type are likely ancestral for red algae, but may have been lost in a number of lineages such as *C*. *crispus* and *G*. *chorda* (Fig. 3). Zinc-finger domains bind DNA, RNA, protein and/or lipid substrates, with the CCHC-type primarily acting in RNA or single-stranded DNA binding^44^. Three of the four CCA zinc-finger proteins possessed a CCHC zinc finger domain, indicating that these proteins may bind to RNA or single-stranded DNA substrates in *Porolithon*, *Lithothamnion*, and *Lithophyllum*. The zinc finger domain of the *Sporolithon* sequence, however, appears to have diverged from a typical CCHC, indicating a different function of this protein in *Sporolithon*. CCHC domain-containing zinc fingers do not form a monophyletic clade in the tree, indicating that this domain (and presumably its binding capability) can be readily lost (or gained) (Fig. 3).

**Figure 4:**
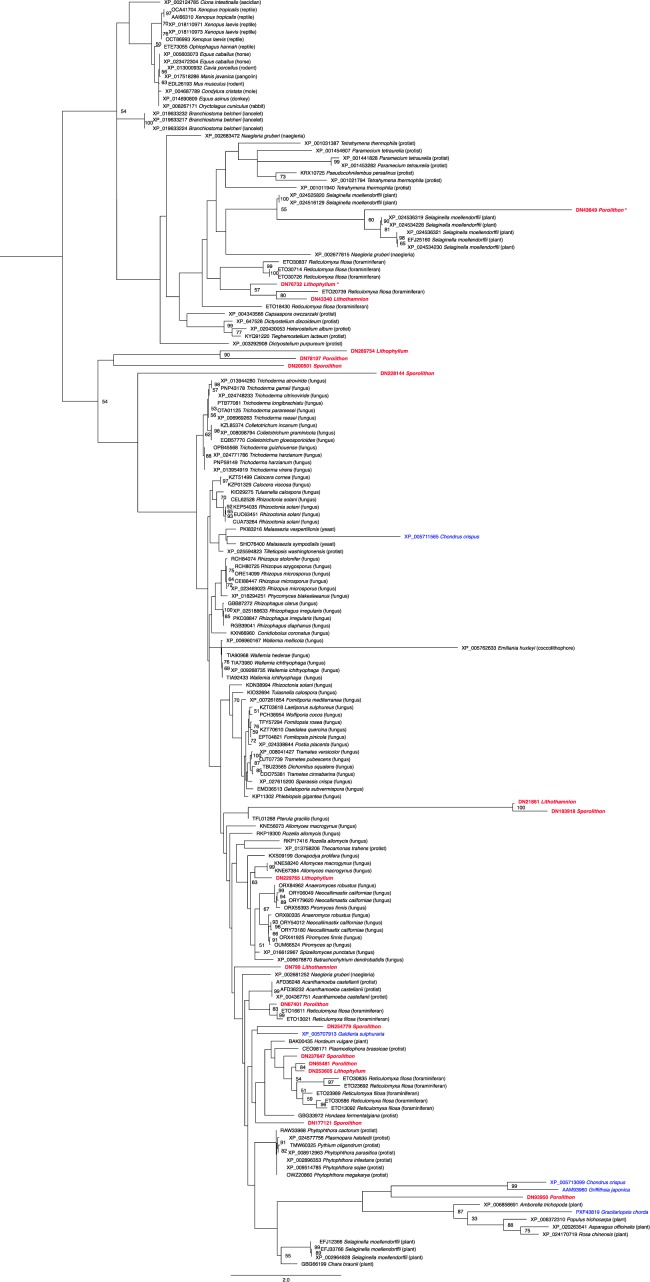
Best scoring maximum likelihood phylogenetic analysis of serine/threonine protein kinase-like sequences. Midpoint rooted tree, with bootstrap values <50 removed. Scale bar indicates the branch length for 2.0 aa substitutions. CCA names are shown in bolded red and other noncalcifying red algae species are in blue. Incomplete sequences that were found within the enriched category but did not have serine/threonine protein kinase-like conserved regions are marked with a*.

Another orthogroup relating to the regulation of transport was found to be overrepresented in CCA when compared to the other two noncalcified species. These genes were primarily from the protein kinase family, specifically, the family of protein kinases that phosphorylate serine/threonine, however, two incomplete sequences possessed conserved unconventional myosin tail domains (marked with * in Fig. 4) but still returned top hits with protein kinase sequences from BLASTP analysis. These sequences were further investigated using the Pfam database^45^, and it was found that there are other proteins that have this domain that are not myosins and that possess the domain arrangement of unconventional myosin tails and protein kinases, specifically serine/threonine protein kinases (STKs). Therefore, it is likely that if these two sequences were full, they would have the conserved STK domain. This gene family appears to have undergone expansions in the CCA lineage, with between 3–6 genes present in each species (Fig. 4). The phylogenetic tree displays the relationship of CCA STKs to those of other eukaryotic species, including algae, plants, and protists. The length of the branches for some of the CCA sequences suggests that these genes are evolving rapidly in CCA, and may also explain why the CCA sequences do not always clade together or with other red algae species. However, support values within this tree are generally low, making reconstruction of evolutionary history difficult. CCA STK-related genes maintained the conserved domains (200–450) that are characteristic of STKs^46^. STKs in plants are described to act as a “central processor unit”, accepting information from receptors that sense environmental conditions and other external factors, and then act to convert those signals into appropriate responses or outputs, such as changes in metabolism, cell growth and division, and gene expression^47^. The number of genes within the STK family could suggest an important role for phosphorylating serine/threonine or phosphoserine/threonine signalling in CCA. An analogous situation occurs in the unicellular green alga *Chlamydomonas reinhardtii*, where the high number (28) of putative tyrosine kinases relates to the importance of phosphotyrosine signalling in this taxon^48^. STKs have only been described in two other multicellular red algal species, with *G*. *chorda* only having one protein identified as an STK. However, the widespread red algal species *C*. *crispus* has a large number of possible STKs in its genome, suggesting this protein family could be important for species that live in variable environments, allowing them to have a more advanced system in responding and reacting to external factors and environmental conditions.

**Figure 5:**
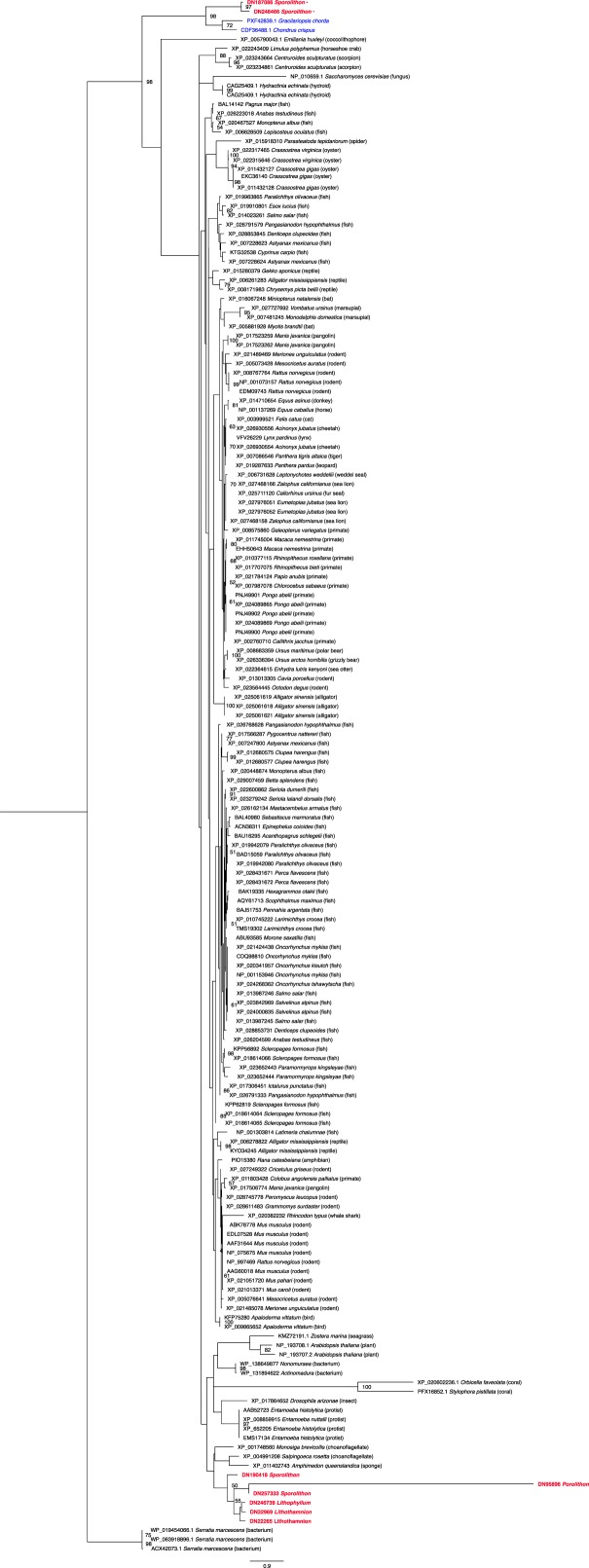
Best scoring maximum likelihood phylogenetic analysis of GH18 like sequences. The clade containing homologous chitinase related proteins from the bacterium *Serratia marcescens* was used as the outgroup. Bootstrap values <50 removed. Scale bar indicates the branch length for 0.9 aa substitutions. CCA names are shown in bolded red and other noncalcifying red algae species are in blue. ^•^Denotes additional CCA GH18 sequences that were not found within the ‘enriched’ category.

Six genes across the four species were found to form a supported clade exhibiting similarity to glycosyl hydrolase family 18-like (GH18) proteins (Fig. 5). The GH18 gene family is previously undescribed in corallines and is generally undescribed in red algae species, although similar proteins from other red algal species contain GH18 conserved regions (PXF42839.1 *G*. *chorda*, CDF36488.1 *C*. *crispus*). In initial phylogenetic analyses the CCA sequences formed a well-supported clade that was not sister to other red algal GH18 proteins (data not shown). Although these noncalcifying red algae (fleshy, temperate species) are phylogenetically and physiologically distant from the CCA investigated here, meaning that, due to functional divergence, their sequences may not always form monophyletic clades, noncalcifying red algal GH18 sequences were used as queries in BLAST searches against the transcriptomes of the four species of CCA to determine if additional GH18 sequences were present. Two additional GH18 sequences were identified from *Sporolithon* with e-values of 0.0 and were added to the analysis (denoted with •, Fig. 5). These two sequences grouped with the other red algal sequences with high support, whereas the six CCA GH18 sequences identified in the enrichment analysis formed a separate clade. It therefore appears that GH18 sequences have duplicated within the CCA lineage, with the duplicates perhaps evolving different functions. Proteins within the GH18 family have been proposed to play a role in polysaccharide processing^49^ and can be active chitinases^50^. Chitin has been described among the polysaccharides of a coralline alga species, *Clathromorphum compactum*, which is an arctic and subarctic species^51^. These chitin containing polysaccharides might be important molecules in the calcification process of this species, with the chitin providing additional strength and protection to the calcified skeleton of *C*. *compactum*, therefore making it more resilient to the negative effects of ocean acidification, or the decrease in ocean pH^51^. GH18 proteins have also been linked to the biomineralization process in the pearl oyster, *Pinctada fucata*^52^, raising the possibility that enriched GH18 like proteins in CCA may similarly play a role in their biomineralization process. It is noteworthy that novel glycosyl hydrolases may be present in CCA whereas, from BUSCO results, highly conserved glycosyl transferases are missing across CCA and other red algal species compared here. This indicates that polysaccharide metabolism may be highly modified within the Rhodophyta.

#### ‘Supramolecular fibre organisation’ related genes

**Figure 6:**
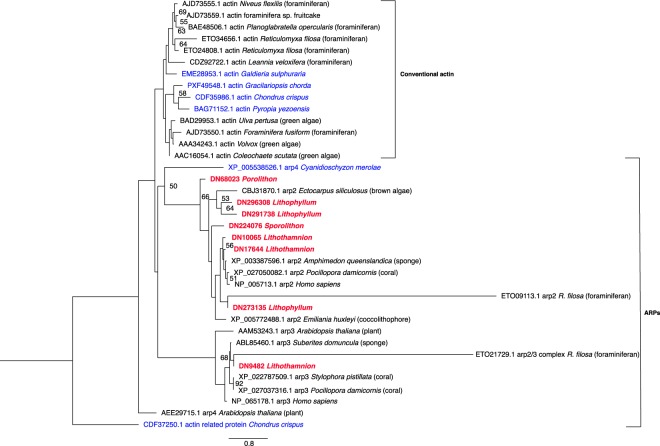
Best scoring maximum likelihood phylogenetic analysis of actin related protein sequences. Tree was rooted at actin related protein (ARP) from *C*. *crispus* (CDF37250.1). Bootstrap values < 50 removed. Scale bar indicates the branch length for 0.8 aa substitutions. Brackets group proteins that are conventional actin sequences or ARPs. CCA names are shown in bolded red and other noncalcifying red algae species are in blue.

The category ‘supramolecular fibre organisation’ was found to be overrepresented in the CCA-specific orthogroups. When investigated further, sequences within this category were found to be actin-related proteins (ARPs) or heat shock proteins (HSPs). A tree was constructed with conventional actin genes (obtained from NCBI) and different families of ARPs from different species (Fig. 6), using ARP 1, 2, 3, and 4 sequences from the evolutionary analysis of the actin family in Goodson & Hawse, 2002^53^. Albeit with low support, it was found that most ‘CCA-specific’ proteins from this family were most closely related to ARP2, however, one protein from *Lithothamnion* was placed within the ARP3 clade. ARPs are found in other red algae species; however, few ARP2 sequences have been found, and no ARP3. Conventional actin in Florideophyceae has been reported to have undergone a duplication event, which is possibly linked to the complexity of thallus organisation and modes of reproduction for this class of algae^54^. Corallines belong to the class Florideophyceae, however no conventional actins were found within their transcriptomes. It is possible that the duplication of ARPs within this lineage created functional redundancy, leading to the loss of conventional actins in CCA, alternatively, conventional actins may be present in CCA genomes but not expressed in the stages sampled here.

Actins are known to play a role in biomineralisation in some unicellular calcifiers. For example, in the calcifying coccolithophore *Coccolithus braarudii* it was found that disruption of the actin network inhibits elements of secretion and biomineralization^55^. More recently, Tyszka, *et al*. (2019) found that F-actin (filamentous actin) is involved in the formation of the calcified chamber/shell of the foraminifera species *Amphistegina lessonii*, ultimately controlling mineralisation^56^. Therefore, it is possible that the expansion of ARP genes found in CCA is associated with the evolution of calcification in this lineage. CCA ARPs do not group with those of calcifying foraminifera in the phylogenetic tree, however given that CCA calcification evolved independently from that of coccolithophores and foraminiferans it is not expected that orthologous actin genes would necessarily be involved in the calcification process of these taxa.

**Figure 7:**
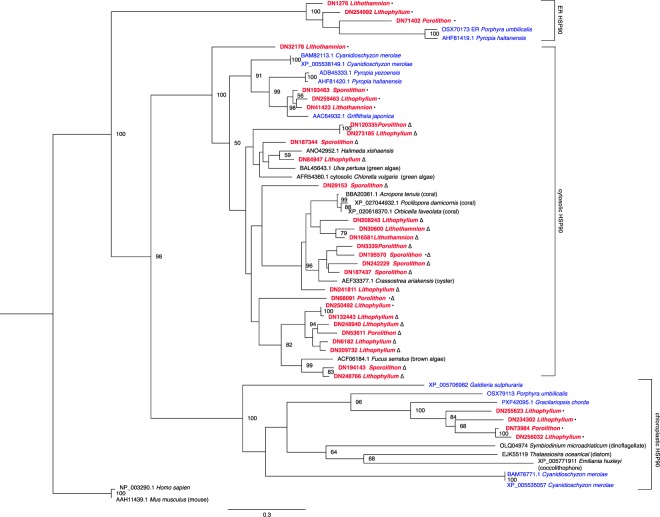
Best scoring maximum likelihood phylogenetic analysis of heat shock protein 90 sequences. Clade containing *Mus musculus* and *Homo sapiens* endoplasmic reticulum (ER) HSP90 was used as the outgroup. Bootstrap values <50 removed. Scale bar indicates the branch length for 0.3 aa substitutions. CCA names are shown in bolded red and other noncalcifying red algae species are in blue. ^•^Denotes HSP90 genes found in CCA that were highly similar to *G*. *chorda* (PXF42095) chloroplastic HSP90 sequence from BLASTP transcriptome analysis. ∆denotes HSP90 sequences that were within the enriched category of ‘supramolecular fibre organisation’. ^•^∆Denotes sequences that were returned from both analyses.

Heat shock protein 90 (HSP90) was also found within the overrepresented category ‘supramolecular fibre organisation’. Phylogenetic analysis reveals that the CCA HSP90 gene family appears to have undergone significant gene duplication events in comparison to other red algae (Fig. 7), and that some duplications likely occurred after the divergence of the four CCA lineages (for example, in *Lithothamnion* and *Sporolithon*, the two earliest-diverging coralline lineages investigated). HSP90 family members have gone through multiple duplication events throughout their evolution and subsequent losses, and can be found throughout different components of a cell^57^. In initial phylogenetic analyses all CCA HSP90 sequences fell within a well-supported clade of cytosolic HSP90 sequences, whereas noncalcified red algae also possessed chloroplastic and endoplasmic reticulum HSP90 genes (data not shown). To determine whether additional HSP90 sequences were present in CCA transcriptomes, the chloroplast HSP90-5 sequence from *G*. *chorda* (PXF42095.1) was used as a query in a BLAST search against the transcriptomes from the four species of CCA. With an e-value cutoff of 1e^−80^, 3–12 additional HSP90 genes per CCA species were identified, including three from *Lithophyllum* and one from *Porolithon* that grouped with chloroplastic HSP90s, and two from *Lithophyllum* and one from *Porolithon* that grouped with endoplasmic reticulum HSP90s in the phylogenetic analysis (Fig. 7). Only 2 sequences were found both within the enriched category of ‘supramolecular fibre organisation’ sequences, and by protein BLAST on the CCA transcriptomes. Overall, there has been extensive duplication of cytosolic HSP90 genes in the CCA species investigated here, most of which likely occurred prior to the diversification of these lineages. These duplications could be linked to the evolutionary history of these algae, having persisted during times of previous elevated temperature^21^. It is also possible that some of the duplications, particularly more recent ones, could be associated with adaptation to particular habitat types (i.e. reef flats or intertidal zones).

#### ‘Adhesion’ related genes

**Figure 8:**
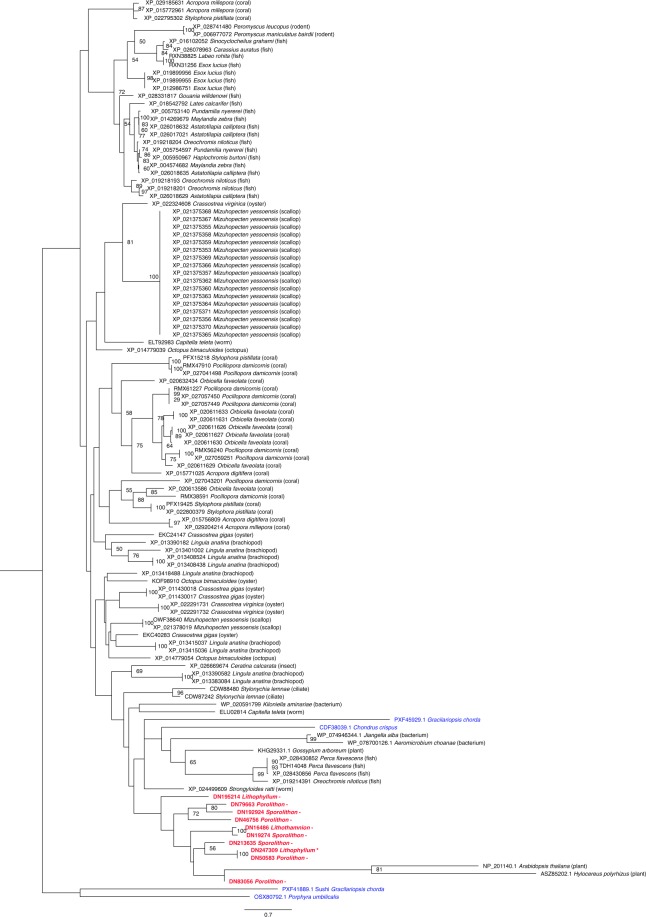
Best scoring maximum likelihood phylogenetic analysis of von Willebrand A domain sequences. The clade containing the sushi domain sequence of *G*. *chorda* was used as the outgroup. Bootstrap values <50 removed. Scale bar indicates the branch length for 0.7 aa substitutions. *Denotes full protein sequences that are likely to be secreted, and therefore extracellular. ^—^Denotes incomplete or partial sequences. All other sequences in the tree are predicted to be intracellular and/or not secreted. CCA names are shown in bolded red and other noncalcifying red algae species are in blue.

The ‘adhesion’ GO term was enriched across CCA-specific orthogroups, potentially relating to their habit as encrusting organisms where cell-cell adhesion and extracellular matrix are essential for maintenance of the structural and functional integrity of the crust. The majority of the proteins within this category returned only hypothetical protein matches or no matches within the Pfam^45^ database, indicating these genes are likely to be unique to CCA. However, across the CCA species, 10 genes contained conserved regions similar to von Willebrand factor type A (VWA) domains found in other eukaryotes (Fig. 8). Intracellular VWA domain proteins common to all eukaryotes are involved in fundamental cellular functions (e.g. transcription ribosomal transport, DNA repair, and protein degradation)^58^. VWA domain proteins in plants and fungi are intracellular, whereas an expansion event in other eukaryotes resulted in large numbers of extracellular VWA domain proteins^58^. The functions of these extracellular VWA domain proteins include cell adhesion and protein-protein interactions, however in molluscs extracellular VWA proteins have been implicated in the formation of calcium carbonate shells^52,59^. VWA domain proteins from CCA appear to form their own, well supported clade away from other intracellular sequences used in this tree (Fig. 8). Most CCA sequences were found to be partial or incomplete, however, one CCA sequence was found to be full-length and is predicted to be secreted, through signal peptide analysis (denoted with * in Fig. 8). Although the majority of CCA sequences were found to be incomplete, the sequences grouped together with the predicted extracellular CCA sequence, therefore it is likely that these enriched sequences are all extracellular (Fig. 8). Therefore, it is possible, that extracellular VWA domain proteins in CCA may have evolved independently in corallines and play a similar role to that of extracellular VWA proteins in other marine calcifiers.

## Conclusions

The ability for coralline algae to deposit high Mg calcite in their cell walls is unique within red algae, as are the intricate calcium carbonate skeletons that allow them to play crucial roles across tropical coral reefs worldwide. From the investigation into the transcriptomes generated in this study, we provide insight into what sets CCA apart from other red algal species, that is, the large number of genes relating to regulation of transport, supramolecular fibre organisation, adhesion, and potentially calcification (e.g. actin related genes and GH18). Our study also offers insights not only into the evolution of coralline algae but more broadly of the red algae (e.g. by confirming the loss of genes in the group). The role of CCA on tropical reefs is integral to reef survival, yet prior to this study, there was limited molecular information for corallines and no complete molecular information for any species of CCA. CCA may be crucial when it comes to the longevity of coral reefs in the face of future environmental change due to their ability to cement and support the carbonate framework of reefs, and for the settlement of important coral reef larvae. This study provides a foundation for future studies of gene expression and function in CCA.

## Methods

### Algae collection

Fragments of CCA ranging in size from 4 cm^2^ to 6 cm^2^ were collected from sites within the lagoon of Lizard Island (Great Barrier Reef, Australia) on SCUBA using a hammer and chisel. Care was taken when collecting to minimise any impact on the reef, and collection was spread out across reefs. Species from low light environments, *Sporolithon* cf.* durum* and *Lithothamnion* cf. *proliferum*, were collected at around 7 m depth from reefs between Bird Islet and Lizard Head. *Porolithon* cf. *onkodes* and *Lithophyllum* cf. *insipidum* were collected from the reef crest, >3 m depth, between South Island and Palfrey Island. A total of 18 individuals from each species were collected over three days, avoiding collection of highly visibly epiphytised fragments. All fragments were thoroughly cleaned, by using a scrubbing brush and razor underneath a microscope, of epiphytes directly after collection and twice more before sampling for molecular analysis.

To ensure the most comprehensive transcriptomes possible and the utility of this dataset for future investigation of CCA response to environmental change, samples were either taken directly after collection from the field, after maintenance for 2.5 weeks under altered pH and temperature conditions, or after maintenance for 3 weeks under common-garden conditions (see Supplementary Methods 1 and Supplementary Table 1). Treatments were conducted at Lizard Island Research Station (LIRS) on Lizard Island from September 2017 to October 2017. The experiment ran for 2.5 weeks, from September to October 2017. All four species of CCA were used within the treatments.

### Molecular sampling

Prior to sampling, each fragment of algae was thoroughly rinsed with filtered seawater and blotted with a kimwipe to remove bacterial film^60^, and then scraped using new, sterile razors into a pre-labelled 1.5 mL microcentrifuge tube containing 1 mL of RNAlater. Care was taken to only remove the top, pigmented, living layer of the CCA, avoiding epiphytes, endolithic algae (which do not penetrate the pigmented layer of the algae and rather sit within the unpigmented CaCO_3_ skeleton), and to eliminate cross contamination between species and treatments. Tubes containing CCA material in RNAlater were then kept at −20 °C at LIRS until being transported on ice to a −20 °C freezer at Griffith University where they were stored until further analysis. CCA host an array of epibionts, and although care was taken in sampling, it is possible that there was some residual contamination.

### RNA extraction

RNA was extracted from CCA samples using a modified TRIzol® RNA extraction protocol from Invitrogen. CCA samples were removed from RNAlater and then homogenised in Trizol (1 mL) at room temperature for 6 mins at 30 Hz using a QIAgen TissueLyser. After the initial 3 mins of homogenisation, samples were removed, placed on ice for 5 mins, and then homogenised for the remaining 3 mins. Further processing followed the manufacturer’s protocol, using bromochloropropane for phase separation, and high-salt solution for precipitation. RNA pellets were resuspended in DNase/RNase-free distilled water (20 µl). Total RNA quantity was determined spectrophotometrically using Invitrogen Qubit® Broad Range RNA kit. RNA yield ranged from 5.36 ng RNA/µl to 800 ng RNA/µl. Presence of contaminating DNA was checked randomly in samples using an Invitrogen Qubit® DNA High Sensitivity kit and returned readings “too low for detection”.

### Library construction and sequencing

RNA samples from all conditions (treatments, field, common garden) were pooled for each species (*n* = 4) prior to sequencing library construction. When pooling samples, similar quantities from each sample was added to the pool (i.e. samples that resulted in high yields were diluted down to match lower yielding samples). Total RNA quantity of pooled samples was checked using the Qubit® to ensure a value of at least 20 RNA/µl; final concentrations for *Sporolithon*, *Porolithon*, *Lithothamnion*, and *Lithophyllum* were 25.4 RNA/µl, 38.6 RNA/µl, 24.4 RNA/µl, and 35.6 RNA/µl, respectively. Quality of pooled RNA was tested using the 4200 TapeStation System. Once RNA was checked, pooled RNA samples (60 µl) were then precipitated and sent to Macrogen, Inc (Seoul, South Korea) for cDNA library preparation using a TruSeq Stranded mRNA LT Sample Prep Kit. The kit used to prepare libraries uses oligo-dT beads to capture RNA species containing polyadenylated tails, therefore minimal bacterial sequences should be present in the resulting transcriptomes. Individually barcoded libraries were sequenced using 100 bp paired-end reads on an Illumina HiSeq 2500 to generate between 87 M and 66 M raw reads per library, with GC content ranging from 45–48%.

### Transcriptome assembly and optimisation

All bioinformatic analyses were performed on Griffith University’s High Performance Computer Cluster “Gowonda”. Quality control was performed on the raw sequence data using FastQC (v 0.11.3, Babraham Bioinformatics). Raw sequences were aligned to assembled transcripts using bowtie^61^ (v 2–2.0.2) and assembled with Trinity^62^ (v 2.4.0) using the default parameters and enabling trimmomatic^63^, to quality trim reads, jaccard clip (which is used for compact genomes), and without normalisation of the reads. To remove redundant transcripts, highly similar sequences were clustered using CD-Hit^64^ (v 4.6.6) using a nucleotide identity threshold of 0.95. To assess assembly completeness, BUSCO (Benchmarking Universal Single-Copy Orthologs)^34^ (v 3.6.1) and the eukaryota_odb9 dataset were used to compare transcriptomes against highly conserved eukaryote orthologs selected from OrthoDB^65^ (v 9.1). Results from BUSCO were compared against whole genome data from the noncalcifying red algae *Chondrus crispus* (PRJNA193762)^43^ and *Gracilariopsis chorda* (PRJNA361418)^66^.

Annotation of the transcriptomes was conducted using Trinotate v 3.1.1^38^, which performed sequence homology searching against the SwissProt database^67^ by BLAST^68^, Pfam database^45^ by hmmscan^69^, and association with Gene Ontology (GO) terms^70^.

### Gene ontology and enrichment analysis

All four transcriptomes contained a large number of redundant transcripts. To reduce this, Transdecoder was first used to identify the single best copy of each transcript, as determined by Transdecoder (http://transdecoder.github.io) using the -single_best_orf command. The dataset containing the single best copy of each transcript was then further filtered to obtain the single longest isoform per Trinity gene. Orthofinder^71^ (v 2.2.3) was then used with this dataset to identify orthologous genes/proteins across CCA species and the whole genome protein data from two other noncalcifying red algae species *C*. *crispus* and *G*. *chorda*. Unique and shared orthogroups were identified using the micropan^72^ (v 1.2), dplyr^73^ (v 0.8.0.1), and tibble^74^ (v 2.1.1) packages within RStudio (v 1.1.456) and visualised using the R package UpSetR^75^.

Enrichment analysis of CCA-specific orthogroups identified from the orthology analysis was conducted using the Cytoscape^39^ (v 3.7.1) plugin BiNGO^40^. This was carried out using a hypergeometric statistical test on GO categories from ‘biological processes’, set with a p value of 0.01. The annotation files for each species of CCA were used, and only groups that were found to be enriched across all CCA species were investigated further.

### Phylogenetic analysis

Proteins within enriched categories were analysed via BLASTP^68^ and homology searching within the HMM database^69^. For sequences returning eukaryotic BLAST hits with significant e-values (1e^−3^) on NCBI’s nonredundant database, once conserved protein regions and associated potential functions were identified, related genes from other species were downloaded from NCBI, focusing predominately on top BLAST hits and supplemented with other algal or plant groups. In some instances (phylogenetic analysis of GH18 and HSP90) sequences from other red algae that maintained similar conserved regions were blasted against the transcriptomes of the four species of CCA and sequences that had e-values close to 0.0 were also used in the phylogenetic trees. Sequences were aligned in AliView^76^ (v 1.23) and phylogenetic analysis was performed using RAxML^77^ (v 8.2.11) with automatic model selection and 100 nonparametric bootstrap replicates. Protein trees were visualised using FigTree^78^ (v 1.4.4).

## Supplementary information

Supplementary Information

## Data Availability

Raw data has been deposited in NCBI under BioProject PRJNA518156. The Transcriptome Shotgun Assembly Fasta files have been deposited at DDBJ/EMBL/GenBank under the accession numbers GHIN00000000, GHIO00000000, GHIP00000000, and GHIV00000000 for *Sporolithon* cf. *durum*, *Porolithon* cf. *onkodes*, *Lithothamnion* cf. *proliferum*, and *Lithophyllum* cf. *insipidum*, respectively.
