# Supplementary material for: De novo transcriptome assembly for four species of crustose coralline algae and analysis of unique orthologous genes
Source: Sci Rep. 2019 Aug 30;9:12611. doi: 10.1038/s41598-019-48283-1 (PMC6717203; doi:10.1038/s41598-019-48283-1)
Supplement: Supplementary file 1 — Supplementary Information [file 41598_2019_48283_MOESM1_ESM.pdf]

De novo transcriptome assembly for four species of crustose coralline algae and analysis of unique orthologous genes.

Tessa M. Page[1,2], Carmel McDougall[1,2], Guillermo Diaz-Pulido[1,2*]
[1]School of Environment and Science, Griffith University, Brisbane, Queensland, Australia
[2]Australian Rivers Institute, Griffith University, Brisbane, Queensland, Australia

Supplementary Information

Supplementary Methods

Experimental setup.

In order to obtain a full profile of genes expressed during natural conditions and altered environmental conditions, we subjected the CCA fragments to a 2.5-week multi factorial experiment, manipulating temperature and CO_2. An outdoor 2-header sump (275 L each) system with twelve 10 L individual treatment tanks was set-up in the outdoor laboratory space at LIRS. pCO_2 was regulated in the two header sumps at ambient (pH 8.10; ~ 400 μatm) and high (pH 7.70; ~1200 μatm) levels and each individual treatment tank at either ambient (27ºC) or high (+ 1.5ºC) temperature. The high pCO_2 and temperature levels represent RCP6.0 scenario for predicted end of century levels[1]. Three individual tanks were allocated for each temperature and pCO_2 combination. Temperature levels were regulated within each treatment tank using 50W Eheim aquarium heaters (EHEIM, Deizisau, Germany). Seawater pH/pCO_2 was controlled by injection of pure CO_2 gas controlled by a pH sensor (Aquarium Controller Evolution, Aquatronica, Reggio Emilia, Italy) within each header sump. Water from the sumps was then distributed (12 L hr^{-1}) to one of twelve treatment tanks matching respective temperature treatment. Each 10 L treatment tank contained one individual from each of the four species used during this study. Temperature and pCO_2 were gradually increased over 48 hours until target temperature (+ 1.5ºC) and pH (7.75) were reached. Following that, individuals were held at constant experimental treatment conditions for two weeks. Algae fragments were gently brushed every 4 – 5 days to rid them of epiphytes. Mortality or changes in health were monitored throughout the 2.5 – weeks.

Seawater measurements.

Salinity and temperature were measured daily by use of a conductivity meter (SevenGo, Mettler Toledo). pH was measured every 30 s within the sumps using a temperature compensated, pH electrode (inPro4501VP, Mettler Toledo) that was calibrated to NBS buffers. pH was monitored within the treatment tanks using an NBS calibrated portable pH meter and electrode (Plug & Play glass pH electrode, Mettler Toledo). Irradiance was measured every other day, with average light level values in shaded regions of the tanks at ~ 30 μmol photons m^{-2} s^{-1} and for the non-shaded areas of the tanks at ~ 200 μmol photons m^{-2} s^{-1}. Alkalinity samples were taken twice during the experiment, by collecting water samples every 6 hours for 24 hours. Samples were collected from every treatment tank and sump and preserved by adding 20 μl of $HgCl_2$. Total alkalinity was measured by potentiometric titrations[2]. Samples were run in triplicate or further until a maximum of 1% error was reached between each replicate. Seawater carbonate chemistry (Table S1) was determined using CO_2SYS v. 2.1[3] with the dissociation constants of Mehrbach et al. 1973[4] refit by Dickson & Millero (1987)[5].

Supplementary Table 1. Values (means ± SE) for carbonate chemistry parameters for each treatment over the course of the 2.5-week experiment. Treatments ACOAT, ACOHT, HCOAT, and HCOHT equate to ambient CO_2 and ambient TºC, ambient CO_2 and +1.5ºC, elevated CO_2 and ambient TºC, and elevated CO_2 and +1.5ºC, respectively. pH is on NBS scale, Ω_{Ca} is equal to the saturation state of calcite, and Ω_{Ar} is equal to the saturation state of aragonite. N = 16.

Treatment	ACOAT	ACOHT	HCOAT	HCOHT
Temp (ºC)	27.13 ± 0.050	28.66 ± 0.110	26.95 ± 0.110	28.57 ± 0.100
Salinity (psu)	34.88 ± 0.050	35.09 ± 0.040	34.94 ± 0.040	35.12 ± 0.030
pH	8.19 ± 0.004	8.18 ± 0.004	7.76 ± 0.003	7.76 ± 0.005
TA (μmol kg^{-1})	2369.55 ± 8.670	2357.52 ± 7.170	2363.57 ± 6.900	2367.65 ± 7.460
pCO_2 (μatm)	406.26 ± 4.401	410.71 ± 4.700	1281.17 ± 11.080	1283.09 ± 15.350
Ω_{ca}	5.66 ± 0.050	5.81 ± 0.060	2.46 ± 0.020	2.62 ± 0.030
Ω_{Ar}	3.75 ± 0.030	3.87 ± 0.040	2.63 ± 0.010	1.75 ± 0.020

References

1 IPCC. Climate Change 2013: The Physical Science Basis. Contribution of Working Group I to the Fifth Assessment Report of the Intergovernmental Panel on Climate Change. 1535 (Cambridge, United Kingdom and New York, NY, USA, 2013).

2 Allan, B. J., Miller, G. M., McCormick, M. I., Domenici, P. & Munday, P. L. Parental effects improve escape performance of juvenile reef fish in a high-CO2 world. *Proc Biol Sci* **281**, 20132179, doi:10.1098/rspb.2013.2179 (2014).

3 MS Excel Program Developed for CO_2 System Calculations (Oak Ridge, Tennessee, 2006).

4 Mehrbach, C., Culberson, C., Hawley, J. & Pytkowicx, R. Measurement of the apparent dissociation constants of carbonic acid in seawater at atmospheric pressure[1]. *Limnology and Oceanography* **18**, 897-907 (1973).

5 Dickson, A. G. & Millero, F. J. A comparison of the equilibrium constants for the dissociation of carbonic acid in seawater media. *Deep Sea Research Part A. Oceanographic Research Papers* **34**, 1733-1743, doi:https://doi.org/10.1016/0198-0149(87)90021-5 (1987).
